# Clinical diagnostic value of viable *Schistosoma japonicum* eggs detected in host tissues

**DOI:** 10.1186/s12879-017-2362-4

**Published:** 2017-04-04

**Authors:** Kongzhen Gu, Yuesheng Li, Patrick Driguez, Qingren Zeng, Xinlin Yu, Hui Sun, Liting Cai, Yongkang He, Wenyang Wang, Donald P. McManus

**Affiliations:** 1 Department of Parasitology, Xiangya School of Medicine, Central South University (CSU), 410013, Tongzipo Road 172#, Changsha, Hunan People’s Republic of China; 2 Molecular Parasitology Laboratory, Queensland Institution of Medical Research, Brisbane, QLD Australia; 3 Xiangyue Hospital, Schistosomiasis Control and Prevention Institution of Hunan Province, Yueyang, People’s Republic of China

**Keywords:** *Schistosoma Japonicum*, Vitality of eggs, Alp, CalS, Aos, Sdhg, SjR2mRNA, Morphological characteristics of eggs

## Abstract

**Background:**

*Schistosomiasis*, one of the neglected tropical diseases, is endemic in more than 70 countries. However, the clinical diagnosis of patients with a low degree of infection is an unsolved technical problem. In areas endemic for *schistosomiasis japonica,* proctoscopy detection of eggs has been one method used for clinical diagnosis. However, it is often a challenge to find typical live eggs and it is difficult to distinguish live eggs from large numbers of partially degraded and/or completely degraded eggs within colon biopsy tissue. To address this problem, we tested six different morphological and biochemical/molecular markers (ALP; morphological characteristics of egg; CalS (calcified substance); AOS (antioxidase); SDHG (succinic dehydrogenase) and SjR2 mRNA (retrotransposons 2 of *S.japonicum* genome mRNA)), including four new markers (CalS; AOS; SDHG and SjR2 mRNA.), to determine the viability of *S. japonicum* eggs deposited in human and mouse colon tissues. Our ultimate aim is to obtain a new method that is more sensitive, practical and accurate to clinically diagnose schistosomiasis.

**Methods:**

Tissue samples were collected from mice at six different time points during *S. japonicum* infection with or without treatment with praziquantel (PZQ). Four new biochemical or molecular markers were used for the detection of egg viability from mouse liver and intestinal samples: CalS; AOS; SDHG and SjR2 mRNA. Subsequently, all markers were employed for the detection and analysis of eggs deposited in biopsy materials from patients with suspected schistosomiasis japonica for clinical evaluation. Microscopic examination of the egg morphology, worm burden in vivo and ALP (alkaline phosphatase) levels were used as a reference standard to evaluate the sensitivity and reliability of four new markers detecting egg viability.

**Results:**

The results of the study showed that the morphology of *S. japonicum* eggs deposited in tissues of hosts with schistosomiasis, especially cases with chronic schistosomiasis, is complex and egg viability is difficult to judge morphologically, particularly eggs with a fuzzy structure or partially modified eggs. We found that the majority of the viable schistosome eggs determined by four new markers (CalS, AOS, SDHG and SjR2 mRNA) were morphologically difficult to identify.

**Conclusions:**

Among the markers, the most sensitive and specific method was the detection of SjR2 mRNA and the most simple, rapid and practical method was the detection of SDHG. Therefore, the detection of SDHG is the most practical for clinical application and its use could improve the accuracy in diagnosing active schistosome infection.

**Electronic supplementary material:**

The online version of this article (doi:10.1186/s12879-017-2362-4) contains supplementary material, which is available to authorized users.

## Background

Schistosomiasis is a disease mainly caused by schistosome eggs lodging and damaging the organs and tissues of the human host. Six schistosome species are endemic in > 70 subtropical and tropical countries, infecting 230 million people and causing 200,000 deaths annually [1–3]. *S.japonicum*, *S. mansoni* and *S. haematobium* are the most common species and schistosomiasis is second only to malaria as a parasitic disease in terms of public health importance. *S. japonicum*, causing zoonotic schistosomiasis, is found only in Asia, and has 60 million people at risk of infection with the potential for further increases in transmission [4]. In China, after sustained control efforts for several decades, the prevalence, morbidity and mortality of the disease has significantly decreased [5]. The alleviation of disease has increased the proportion of chronic cases and asymptomatic carriers with low worm burdens, and increased the technological difficulties in the definitive diagnosis of patients. Additionally, this diagnostic deficiency may delay or prevent the control and eventual elimination of the disease in low transmission areas.

*S. japonicum* and *S. mansoni* have a similar life cycle in their definitive mammalian hosts. Adult worms reside in the mesenteric veins and excrete eggs that primarily circulate to, and lodge in, the liver and intestines. The eggs lodged in the colon submucosa and mucous layers, prior to rupturing into the lumen of the bowel and passing out with the feces, can be used for clinical diagnosis. In China, field and clinical diagnosis techniques for *S. japonicum* are mainly dependent on stool examination of eggs and immunological detection of specific antibodies [6, 7]. Methods of schistosomiasis diagnosis using the detection of eggs include the Kato-Katz thick smear technique, a nylon screen method, the miracidium hatching test and rectal biopsy [8]. However, all stool examinations are time-consuming, laborious, have a high false negative rate [9], and for those chronic patients with low infection burden, fibrosis of the intestinal wall reduces the probability of finding eggs in stool. Consequently, stool examination is not suitable for the diagnosis of patients in endemic areas with low-intensity infections [10]. In contrast, immunological diagnostic techniques are fast, sensitive and convenient, have been used since the early 1980s to estimate infection rates and for screening chemotherapy treated subjects in endemic schistosomiasis areas, and for the last 20 years have been extensively used for clinical and field diagnosis. However, because of cross-reactions with antigens from other parasites and the inability to distinguish a current infection from a previous infection, it is impossible to use immunological tests for the assessment of cure [11, 12]. Therefore, immunological methods are suitable for auxiliary diagnosis but not for a definitive diagnosis. Recently, molecular techniques that detect free schistosome-specific DNA in patient serum or urine samples have been developed [13–16], but have not yet been used in the field or clinic.

Detecting schistosome eggs from rectal biopsy specimens, typically employed in the clinic for the diagnosis of chronic and advanced schistosomiasis, partially remedies the drawbacks of the previously mentioned diagnostic techniques. Nevertheless, there are still some prominent problems with this method. One difficulty is the probability of finding randomly scattered eggs within biopsy tissues. The other difficulty is finding live eggs from a large number of partially degraded and/or completely degraded eggs within colon biopsy tissue. In addition, the use of this technique requires the professional skills of a biopsy specialist [17]. Furthermore, there is no reliable method to definitively diagnose a patient who has a currently active infection when the viability of the eggs in the specimen is unknown. In this study, we focus on methods of differentiating between viable and non-viable eggs in order to improve the accuracy of clinical diagnosis.

The viability of schistosome eggs has been studied using TTC ninhydrin, acridine orange and iodine staining. However, these methods are complicated and none are applicable to the clinic [18–20]. The detection of alkaline phosphatase (ALP) in *S. mansoni* eggs in paraffin tissue sections from the liver and intestine of infected mice has been used by Giboda and Zdarska [21] to determine egg viability, and shown to be a sensitive and specific method for discriminating viable from dead eggs. Promisingly, viable and dead *S. mansoni* eggs were detected in the intestines of mice by endoscopic confocal laser scanning microscopy [22]. This method could potentially be extended to the clinical setting. The viability of *S. japonicum* eggs from perfused rabbit liver can also be determined by the physical characteristics of the eggs [23]. However, detection of these markers is complex or time-consuming and would be difficult to adapt for clinical diagnosis.

Our study was designed to address the limitations of current diagnostic methods for differentiating between viable and non-viable eggs, particularly for application on rectal biopsy samples. To achieve this we used the mouse/*S. japonicum* challenge infection model and collected samples at different time points pre- and post-infection, and before and after praziquantel (PZQ) treatment. Liver and colon tissues were collected as well as information on pathology and parasitological measures such as liver and colon condition, adult worm and egg numbers and egg morphology. These parameters were then compared with five egg viability markers: calcified substance (CalS), antioxidase (AOS), succinic dehydrogenase (SDHG), SjR2 mRNA and alkaline phosphatase (ALP). Subsequently, the egg viability assays were used on proctoscopic samples from schistosomiasis outpatients to evaluate the applicability and practical value of these markers in clinical diagnosis. ALP and CalS used paraffin embedded tissue sections while AOS and SDHG required intact eggs from tissue. Our results indicated that all four new viability indicators, CalS, AOS, SDHG and SjR2 mRNA, could be employed as markers to evaluate the survivability and viability of eggs. Of these, the detection of SjR2 mRNA was the most sensitive, specific and reliable but also technically complex, and the detection of SDHG was the simplest, most practical and amenable to the demands of clinical application.

## Methods

### Experimental schistosomiasis in mice

Forty-two 7–8 week old male and female Kunming mice (Department of Experimental Animals, Central South University) were used for experimental schistosome infections. *S. japonicum* cercariae were released from naturally infected snails collected from the Dongting Lake, Hunan province. The mice were divided into post infection (PI) and post treatment (PT) time course groups. The PI group were percutaneously [24] exposed to 20 ± 1 cercariae/mouse and kept under standard animal housing conditions for 45 days (45 dPI; *n* = 6), 90 days (90 dPI; *n* = 6) and 180 days (180 dPI; *n* = 6) prior to perfusion. The PT group were infected as above and then treated with two consecutive gavage dosages (300 mg/kg/day) of PZQ (Nanjing Pharmaceutical, China, lot Number: 20,110,307) at 45 days post-infection. The PT mice were divided into three groups and maintained for 30 days (30 dPT; *n* = 6), 90 days (90 dPT; *n* = 6) and 180 days (180 dPT; *n* = 6) before perfusion. Another two groups of uninfected mice (*n* = 3) were used as controls.

### Collection methods for testing samples from experimental mouse models

Mice were euthanized and immersed in 75% (*v*/v) ethyl alcohol for 15 min. The pleuroperitoneal cavities were opened under aseptic conditions to expose the livers and the gross morphology was recorded [25]. The mice were perfused with 0.38% sodium citrate saline solution (DEPC-treated water) to collect and count worms from the portal vein and mesenteric vessels before small tissue blocks of livers and intestines were excised and stored in liquid nitrogen for future extraction of mRNA. Liver and intestine samples for biochemical analysis were then collected. The tissue samples were taken from the left liver lobe or colon (within the first 5 cm from the rectum). From each mouse, 25 liver and 25 colon samples were collected and preserved in liquid nitrogen. Samples to be used for the detection of ALP and CalS were fixed with 4% (*v*/v) paraformaldehyde solution for 16 h and stored in PBS at 4 °C. The AOS assay and SDHG staining of intact eggs were carried out within 4 h of sample collection.

### Collection of colorectal mucosa biopsy specimens from schistosomiasis patients

All the colorectal mucosa biopsy specimens were obtained from patients with schistosomiasis in the outpatient service department of Xiangyue Hospital, Schistosomiasis Control and Prevention Institution of Hunan Province, Yueyang, At the time of sample collection the following data were collected: basic patient information, past medical history, history of contact with infectious water, anti-schistosomiasis drug treatment, present symptoms, results of clinical examination, and ultrasonic examination of liver. The data were recorded in tables specifically designed for the current study. Two pieces of colon mucosa retrieved through a routine proctoscopic biopsy were smeared between two glass slides for microscopic observation. The tissues containing schistosome eggs were captured and analyzed with an Image Acquisition System (Olympus BX41microscrope fixed with an Olympus DP71 camera). The samples were divided into batches with different processing for the various marker assays. The first batch of 35 egg-containing samples were collected for detection of ALP and were fixed with 4% paraformaldehyde solution, visualized microscopically and paraffin embedded. A further 33 and 30 samples were collected for the AOS and SDHG assays, respectively, following microscopy. Finally, 48 samples were collected for mRNA assay, 38 of which contained *S. japonicum* eggs. The biopsied samples were immersed in RNA preserve solution (Solarbio Science &Technology Company, Beijing) and then pressed between two DEPC-treated glass slides. Images were recorded, the egg morphology noted, and then the slides were stored in liquid nitrogen.

The eggs were then further classified from the captured images into mature eggs, immature eggs, partially degraded eggs and completely degraded eggs using a published protocol [26]. Some faint yellow eggs with miracidia were difficult to classify; these were coded as an unknown type of egg.

### Staining of ALP and calcium compounds in *S. japonicum* eggs in host tissue sections

Tissue blocks containing *S. japonicum* eggs were fixed, dehydrated and embedded in paraffin by conventional methods. Serial tissue section slices (10 μm) were cut with a Leica (RM2225) microtome, and 38 successive tissue sections per tissue sample were mounted on glass slides. The prepared slides were stored at 4 °C for subsequent ALP staining and detection of calcium compounds.

#### Detection of ALP in eggs (NBT/BCIP staining)

NBT (Nitro-Blue-Tetrazolium, PR1100, Solarbio Science &Technology Company, Beijing) when mixed with BCIP (5-bromo-4-chioro-3- indolyl-phosphate, Solarbio Science &Technology Company, Beijing) and catalyzed by ALP creates detectable blue or purple deposits in histology tissue sections. ALP detection was completed according to the manufacturer’s protocol and our previous study [27]. Briefly, a working solution was prepared from NBT, ALP reaction buffer and BCIP. Mounted tissue sections were dewaxed, hydrated and incubated at room temperature (RT) for 30 min. Staining solution was added for approximately 15–20 min before the reaction was stopped with ddH_2_O. Finally, the slices were sealed with glycerin jelly. False positive reactions were avoided by including a negative control for each staining experiment. The negative controls were dewaxed tissue sections boiled to inactivation ALP. All stained sections were microscopically examined. Viable eggs were defined as those with a strong blue or purple stain relative to the negative control sample. Stained and unstained eggs in each slice were counted and the images captured. The positive rate of stained eggs in a section was calculated.

#### Detection of CalS in eggs (von Kossa staining assay)

The standard procedure for the detection of calcium [28] was followed. Dewaxed paraffin sections were hydrated and washed twice with ultrapure water. Samples were soaked in 1% AgNO_3_ and then exposed to a 30 W UV lamp for 1 h.The negative control slide was left unstained. Samples then washed two times with ddH_2_O to terminate the reaction and re-dyed for 1 min with 1% neutral red. After dehydration of gradient ethanol and sealing slices with neutral gum, slices were observed under a microscope. Eggs partially or completely covered with black calcium granules were considered as negative or dead. Eggs without black calcium granules were identified as positive and therefore viable. The positive and negative eggs were counted and images captured.

### Detection of AOS in intact *S. japonicum* eggs within host tissue (DAB staining)

Antioxidase (AOS) in the form of peroxidase was detected with a modified DAB (3,3-dimethylbenzidine)-H_2_O_2_ substrate reaction system that results in insoluble brown or pale brown granules in the presence of peroxidase; an optimized procedure is available [29]. Fresh tissue samples were pressed between two glass slides for assessment of egg viability. The remaining tissue was placed in DAB-H_2_O_2_ solution and incubated at for 30 min at RT in darkness with shaking. The reaction was terminated with ddH_2_O and the tissue blocks were pressed between two glass slides for microscopic observation. A negative control tissue block was prepared by heat inactivation at 100 °C for 10 min prior to staining. Eggs with brown or deep yellow granules were judged as positive eggs. The positive and negative eggs were counted, images were captured, and the DAB positive rate of each group was calculated.

### Detection of SDHG in intact eggs within host tissue (MTT staining)

**Fig. 1:**
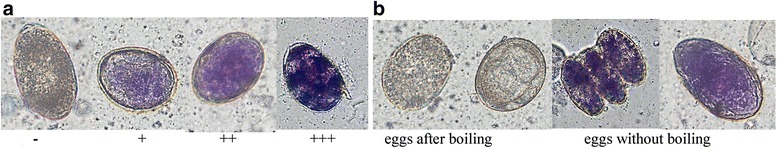
**a** Negatively and positively SDHG-stained eggs. **b** SDHG staining with or without heat inactivation. No color change, negative (−); uneven light purple, weakly positive (+); even purple reaction, positive (++); dark violet, strongly positive (+++)

Succinic dehydrogenase in intact *S. japonicum* eggs was detected using the MTT (Methyl Thiazolyl Tetrazolium; Invitrogen, Carlsbad) assay. The MTT staining reagent (2 g/L in PBS, pH 7.4) was prepared according to the manufacturer’s protocol (Sigma). Fresh tissue samples were pressed between two glass slides and egg types recorded prior to dyeing. The mounted slide was covered with the dye reagent in a humidified box for 30 min.The dye reagent was flicked off and the reaction was terminated with distilled water. The mounted slides were examined microscopically. Egg vitality was classified as weakly positive, positive, strongly positive or negative depending on the degree of staining and previously recorded egg type. Eggs were classified using the following criteria: no color change was negative (−), uneven light purple was weakly positive (+), even purple reaction was positive (++), dark violet was strongly positive (+++) (Fig. 1a and b). The numbers of each type of egg were counted, images captured and the positive MTT staining rates of each group were calculated. A negative control slide was made by heat inactivating tissue for 10 min.

### Detection of specific mRNA from *S japonicum* eggs within hosts

The *S. japonicum* retrotransposon gene, SjR2 [30], was selected as an egg viability marker for detection by real-time PCR. A set of detection primers, forward primer 5′ -ATTGTGCAGCAGTCAGATCC-3′ and reverse primer 5′- ATGCATTGCTTACTCGGTTG -3′, were designed to amplify a 147 bp fragment. For absolute quantification by RT-qPCR an external standard was used [31]. A 408 bp target fragment was obtained by PCR by amplifying an open reading frame (ORF) of SjR2 using forward primer 5′-ACGCGTTTATTGTGCAGCAGTC-3′ and reverse primer 5′- GGGCCCCAACCAGGTAGTCTA-3′(Nanjing Genscript Science & Technology Biology Corp, Nanjing, China). The amplicon was purified by Cycle-Pure Kit (OMEGA Bio-Tek,Norcross GA), following with determination concentration by Nanodrop 2000(Thermo,Beijing). Based on the formula:f Copies/ml = 6.02 × 1023 × ConDNA(ng/μl) × 10^−9^/(DNA length × 660), the number of amplicon copies was calculated, which was diluted into seven standards of 1 copies, respectively.

#### Extraction of RNA and synthesis of cDNA from *S. japonicum* eggs in host tissue

RNA extraction was carried out according to a standardized method [32]. Approximately 50 mg of human or mouse colon mucosa was ground thoroughly in liquid nitrogen and 1 ml Trizol (Invitrogen, Carlsbad) added and left overnight at −80 °C for tissue lysis. After lysis, 200 μl chloroform was added, vortexed and thenthe tube was rested for 2–3 min followed by centrifugation at 12000×g for 15 min at 4 °C. The upper aqueous phase was aspirated and mixed with an equivalent volume of dimethylcartinol, inverted gently and incubated at RT for 10 min. The solution was centrifuged at 12000×g for 10 min at 4 °C and the supernatant discarded. 1 ml of freshly diluted 75% ethanol, made with DEPC treated water, was added and centrifuged at 7500×g for 10 min at 4 °C. The supernatant was removed and the RNA pellet thoroughly air dried at RT. The precipitate was dissolved in 20 μl DEPC treated water and the concentration of RNA was measured. cDNA was generated from 2 μ g of RNA and random hexamer primers (RevertAid First Strand cDNA Synthesis Kit, Thermo Scientific) [33]. After gentle mixing the tube was incubated for 5 min at 65 °C and then chilled on ice. The standard protocol was followed to complete the synthesis of cDNA for real-time PCR assays.

#### Real-time PCR detection of mRNA specific to *S. japonicum* eggs

SjR2 mRNA was detected using a real-time PCR assay based on the SYBR^®^ green fluorescent dye quantitative method (Vazyme AceQ™ qPCR) [34]. A 20 μl reaction mixture was prepared with 10 μl SYBR Green Master Mix, 1 μl of both primers (500 nM), 2 μl of sample cDNA (100 ng/μl) and 6 μl of DEPC treated water. The cycling conditions were: 95 °C for 5 min,followed by 35 cycles of 1 min at 95 °C,1 min at 60 °C,30 s at 72 °C. PCR was performed on a real time thermocycler (Step One Plus Real-Time PCR System, Applied Biosystems).

### Statistical analysis

SPSS 19 statistical software was used to analyze the data. Differences between groups were tested with the non-parametric Anova Kruskal-Wallis H test and the Nemenyi test for pairwise comparisons. The Chi-square test was used for testing the results of each marker compared with different types of eggs. Statistically significant results were defined as *P* ≤ 0.05. The Pearson product moment correlation coefficient r was adopted for correlation analysis.

### Ethics statement

Prior to the commencement of this study, the study protocol was reviewed and approved by the Ethics committee of Hunan Institute of Parasitic Disease (reference number: 2014-S003). Before participating in the study, all subjects were given detailed explanations about the objectives and methodologies of this research. Written informed consents to participate and publish were obtained from all participants who provided colorectal mucosa biopsy samples voluntarily. The study was performed in accordance with the recommendations of the Chinese code of practices for the care and use of animals for scientific purpose. The Ethical Committee of the Center for Parasitology Research (ECCPR) has approved all experimental procedures, including animal handling, under animal license number: syxk (Xiang) 2011–0001 and in accordance with strict ethical standards. Laboratory animal quality conformance license number:43,006,700,002,928.

## Results

### Morphological types of eggs within host tissues

#### Differences in types of eggs within colon tissues collected from mice post infection or post treatment

Five tissue fragments of identical colon segments from each mouse, either in infected groups or in treatment groups, were pressed between glass slides for microscopic examination. The eggs were classified using morphology as immature, mature, partially degraded, completely degraded, and unknown type (Additional file 1: Figure S1). Based on morphology, typical immature or mature eggs (unchanged in structure or color) can be defined as live or viable, and completely degraded eggs (structural disorder or blackened in color) can be defined as dead or non-viable. However, partially degraded and unclassifiable type eggs have unknown viability. Partially degraded and unknown type eggs were present in samples post infection (PI) and post treatment (PT) (Table 1). The numbers and types of eggs deposited in colon tissue did not significantly increase as the time of PI progressed and were not proportional to the number of adult worms. The numbers and types of eggs in mice at 90dPI with 8 worms and those in mice at 180dPI with 6 worms were similar.

**Table 1:** Proportions of egg viability in colon tissues of mice infected with *S. japonicum* and with or without PZQ treatment

		Days post-infection			Days post-treatment with PZQ		
		45d	90d	180d	30d	90d	180d
Mouse numbers		6	6	6	6	6	6
Worm burden	Mean ± SD	17.2 ± 3.3	7.6 ± 1.7	4.5 ± 0.7	0	0	0
	male-female worms pairs	8.4	3.8	1.6	0	0	0
Eggs in colon biopsies (total of 30 biopsies)		4666	4840	4532	4497	2320	1587
The proportion of different types of eggs	Immature eggs (total)*	39.4% (1840)	19.8% (960)	14.7% (666)	1.1% (49)	0	0
	Mature eggs (total)*	51.7% (2412)	34.0% (1644)	20.8% (943)	1.7% (75)	0	0
	Partially degraded eggs (total)	8.9% (414)	11.7% (568)	18.6% (843)	54.2% (2436)	12.8% (296)	11.1% (176)
	Completely degraded eggs (total)	0	29.6% (1433)	35.6% (1613)	18.8% (845)	72.5% (2024)	88.9% (1411)
	Unknown viability type (total)	0	4.9% (235)	10.1% (467)	27.0% (1216)	14.7% (342)	0

* Immature eggs and mature eggs were judged to be live or viable by morphology

As expected, the number of eggs significantly decreased with increasing time PT. The percentage of immature and mature eggs decreased with time PI while unknown viability eggs, partially and completely degraded eggs all increased. Predictably the number of unknown type eggs increased and live eggs disappeared after PZQ treatment and the elimination of adult worms. At 30 days PT, the majority of eggs deposited in host colon were degraded with few live eggs and 27% unknown type eggs. At 90 days PT, nearly all eggs were completely degraded eggs with only a few partially degraded and unknown type eggs evident. Finally, at 180 days PT, 88.9% eggs were completely degraded eggs, the remainder were partially degraded, and neither live nor unknown type eggs could be found.

#### Differences in types of *S. japonicum* eggs from the colon mucosa of schistosomiasis japonica patients

Seventy-six outpatients suffering from schistosomiasis japonica presented at XiangYue Hospital and were included in this study. Colon mucosa samples were collected via proctoscopic biopsy and microscopically examined. Within colon mucous membrane biopsies there was an absence of typical *S. japonicum* immature and mature eggs; however, some potentially viable and unknown type eggs, and numerous partially degraded and completely degraded eggs (Additional file 2: Figure S2) were present. Of specimens collected from 76 patients, 69 contained partially degraded eggs, 73 completely degraded eggs, eggs whose viability could not be determined by microscopic examination were found in 44 specimens, and 32 specimens contained unknown type eggs (Additional file 3: Table S1). The majority of patients had several categories of eggs within their biopsy samples; six patient specimens consisted of only completely degraded eggs.

### Biochemical viability markers for *S. japonicum* eggs

#### Biochemical markers for eggs deposited in liver and intestine from infected mice with or without PZQ treatment

**Fig. 2:**
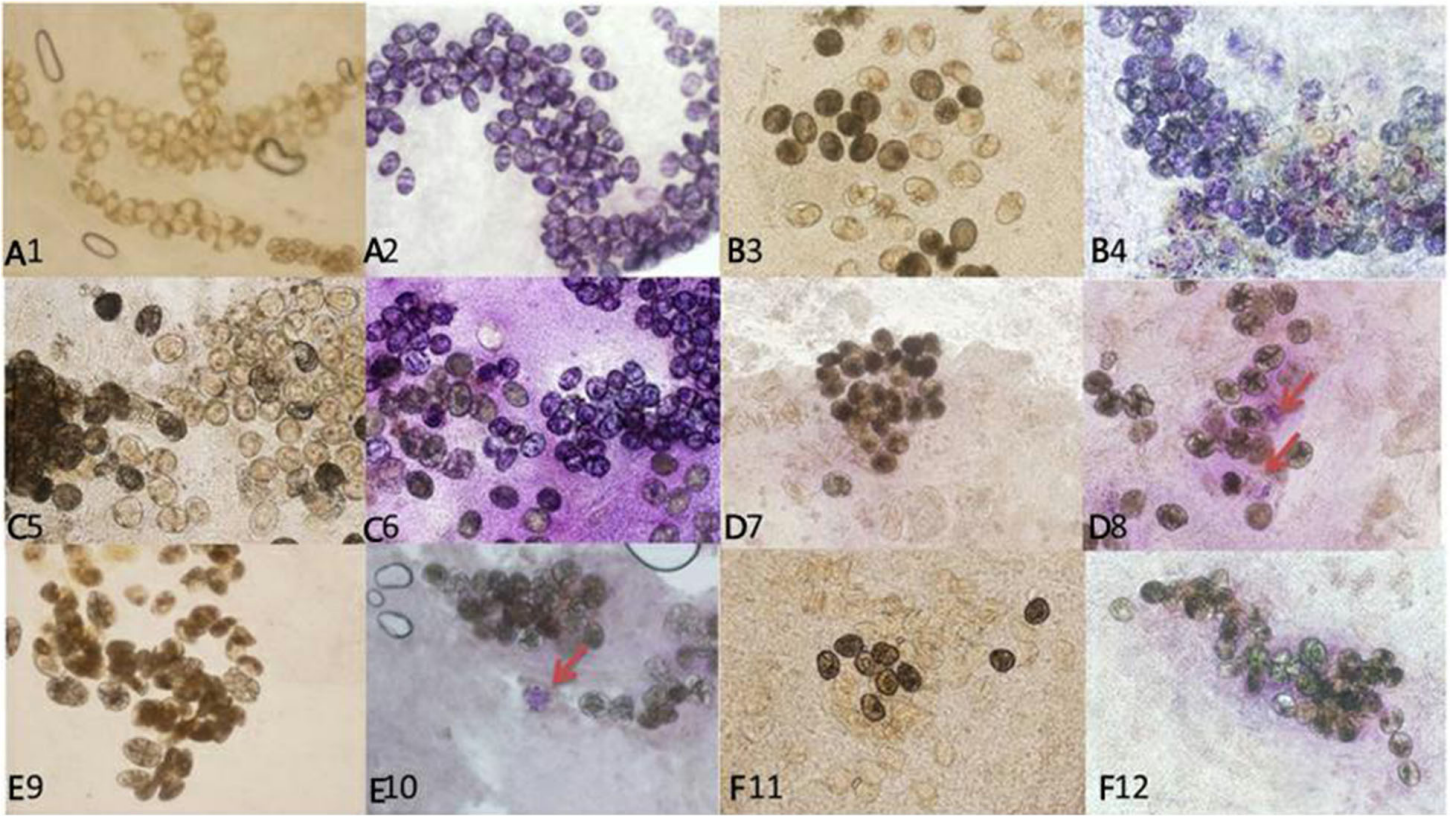
SDHG staining results for *S. japonicum* eggs in mouse colonic tissue. Eggs deposited in colon tissues of mice at 45 days post infection (PI) were light yellow when unstained (A1) and positive eggs ranged from light to dark purple after MTT staining (A2). Eggs deposited in colon tissues of mice 90 days PI were light yellow, light black and black when unstained (B3) and after staining with MTT, most eggs appeared light purple or purple (B4). Eggs deposited in colon tissues of mice at 180 days PI were light yellow, brownish, light black or black when unstained (C5) and following staining with MTT most eggs revealed a positive purple response while a minority were not stained, a negative response (C6). Eggs deposited in colon tissues from mice at 30 days post PZQ treatment were light black and black with a few yellow eggs when unstained (D7); after staining with MTT few eggs were light purple (red arrows), indicating a weak positive reaction (D8). Ninety days PT eggs deposited in unstained mouse colon tissue were only light black and black (E9); after staining with MTT only a few eggs were light purple (red arrow) indicating a weak positive reaction (E10). A hundred and eighty days PT unstained eggs in mouse colon tissue were either empty (yellow) or black (F11) and after staining with MTT no eggs showed a positive color reaction (F12). All images are at ×100 magnification

ALP, SDHG, CalS and AOS were assessed in liver and colon tissues collected from mice post-infection and with or without treatment with PZQ (Additional file 4: Figure S3, Additional file 5: Figure S4 and Additional file 6: Figure S5, Fig. 2 and Tables 2- 3). The rate of positive biochemical viability markers for *S. japonicum* eggs in liver and colon decreased with increasing days post infection (Table 2). Similarly the number of positive eggs in liver and intestine tissue detected by the four biochemical markers significantly decreased with increasing time post PZQ treatment (Table 3). However, ALP, SDHG and CalS staining was reduced more in intestine tissues than in liver tissues at 3 months post treatment onwards (*P* < 0.05; Table 3). In general, the marker positive eggs deposited in liver and intestine tissues had a similar tendency to decrease in infected groups with or without PZQ treatment. Compared with ALP, the sensitivity of AOS was the lowest and SDHG was the highest. Overall, a higher ratio of marker positive to negative eggs appeared in the immature, mature and partially denatured eggs (Fig. 2 and Table 4).

**Table 2:** Comparison of five viability markers in eggs deposited in liver and colon tissue from mice infected with *S. japonicum*

Staining method	Marker	Positive rate (positive/total eggs; %) at different days post infection					
		Eggs in hepatic tissue			Eggs in colon tissue		
		45d	90d	180d	45d	90d	180d
	Viable morphology	90.7 (3857/4253)	54.2* (2510/4631)	35.0* (1511/4318)	91.1 (4251/4666)	53.8* (2604/4840)	35.5* (1609/4532)
NBT/ BCIP	ALP	100.00 (368/368)	82.25 (306/372	48.54 (234/482)	100.00 (287/287)	74.36 (302/406)	66.59 (281/422)
von Kossa	CalS▼	100.00 (460/460)	63.86* (440/689)	43.14 (280/649)	100.00 (378/378)	45.89* (179/390)	35.87* (155/432)
DAB	AOS	92.68 (2192/2365)	54.05* (1308/2420)	42.70 (889/2082)	93.45 (2170/2322)	46.03* (976/2120)	36.28* (755/2086)
MTT	SDHG	98.29 (1607/1635)	60.42* (815/1349)	50.52 (776/1536)	99.19 (1348/1359)	63.71* (741/1163)	59.36 (631/1063)

ALP (alkaline phosphatase), CalS (calcified substance), AOS (antioxidase) and SDHG (succinic dehydrogenase). ▼: Data are non-calcified number of eggs. *: Compared with ALP, *P* < 0.05. All the data were collected from examining 18 blocks of liver and intestinal tissue samples from each of six mice

**Table 3:** Comparison of five markers in eggs deposited in liver and colon tissue from mice infected with *S. japonicum* post treatment

Staining method	Marker	Positive rate (positive / total eggs; %) at different days post treatment with PZQ					
		Eggs in hepatic tissue			Eggs in colon tissue		
		30d	90d	180d	30d	90d	180d
	Viable morphology	2.7* (91/3369)	0.00* (0/2713)	0.00 (0/1548)	2.8* (126/4497)	0.00 (0/2320)	0.00 (0/1587)
NBT/ BCIP	ALP	83.52 (304/364)	34.88 (75/215)	5.83 (6/103)	75.35 (217/288)	0.00 (0/187)	0.00 (0/ 149)
von Kossa	CalS▼	78.57 (176/224)	15.22* (21/138)	2.97 (3/101)	68.75 (143/208)	8.45 (12/142)	2.94 (3/102)
DAB	AOS	13.97** (96/687)	0.00** (0/323)	0.00 (0/312)	19.90** (83/417)	0.00 (0/220)	0.00 (0/126)
MTT	SDHG	11.41** (67/587)	5.70** (29/509)	0.00 (0/456)	7.16** (38 /531)	3.08 (9/292)	0.00 (0/266)

ALP (alkaline phosphatase), CalS (calcified substance), AOS (antioxidase) and SDHG (succinic dehydrogenase). ▼: Data ares non-calcified number of eggs. Compared with ALP: * *P* < 0.05; ** *P* < 0.01. All the data were collected from examining 18 blocks of liver or intestinal tissue samples from each of six mice

**Table 4:** Comparison of the positive rates (%) after staining all types of eggs deposited in tissues of mice with AOS and SDHG either prior or post treatment

Method of staining	Marker	Immature eggs		Mature eggs		Partially degraded eggs		Completely degraded eggs	
		90d PI	90dPT	90d PI	90d PT	90d PI	90d PT	90d PI	90 d PT
DAB	AOS	74.17 (712/960)	0.00 (0/316)	68.85 (1132/1644)	0.00 (0/468)	67.60 (384/568)	0.00 (0/136)	00.00 (0/1648)	0.00 (0/654)
MTT	SDHG	99.11* (446/450)	0.00 (0/0)	97.58* (966/990)	0.00 (0/0)	70.76 (472/667)	3.36 (4/119)	0.00 (0/264)	0.00 (0/513)

MTT: DAB,*:*P* < 0.05

#### Comparison of different viability types of eggs in colon tissues from infected mice with or without PZQ treatment using AOS and SDHG assays

As indicated in Table 4, the positive rate of SDHG detection in morphologically immature (99.11%) and mature eggs (97.5%) was significantly higher than that of AOS (74.14% and 68.85%, respectively; *P* < 0.05). However, for partially degraded eggs, AOS and SDHG as viability markers, were not significantly different (*P* = 0.128).

#### Analysis of three biochemical markers for eggs within rectal mucosa biopsy samples from patients infected with *S. japonicum*

The results of three biochemical markers testing eggs within rectal mucosa samples from schistosomiasis japonica patients showed that a high ratio of positive eggs appeared in suspected viable unknown type and partially degraded eggs (Table 5). The positive rates of ALP, SDHG and AOS, were significantly different (*P*<0.05) compared with the results of the unknown type, suspected viable and partially degraded eggs by morphology.

**Table 5:** Comparison of the egg viability in the colonic mucosa proctoscopy samples from *S. japonicum* patients

Markers	Cases	Egg category in mucosa biopsy				Confirmed diagnosis rate (%) **(**positive cases/ detected cases)
		Suspected viable eggs cases	Unknown type eggs cases	Partially degraded eggs cases	Completely degraded eggs cases	
Morphology	41	6	5	21	9	14.63 (6 / 41)
ALP positive cases/ detected cases	35	5/6	3/5	7/15	0/9	42.86 (15/35)^*^
AOS positive cases/ detected cases	33	5/6	2/5	3/13	0/9	30.30 (10/33)^*^
SDHG positive cases/ detected cases	30	4/4	3/6	6/14	0/6	43.33 (13/30)^*^

*: Compared with morphology, *P* < 0.05

### Analysis of specific mRNA of eggs in liver and intestine tissues from mice and patients infected with *S. japonicum*

**Fig. 3:**
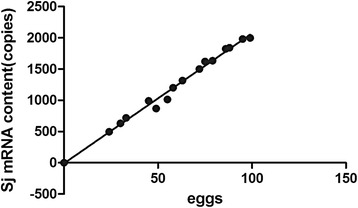
Average content of SjR2 mRNA per *S. japonicum* egg. Regression of the number of *S. japonicum* eggs versus PCR amplified SjR2 mRNA copies per sample

Real-time qPCR was efficient with consistent melt curves and a linear standard curve (Additional file 7: Figure S6). Regression analysis allowed the average copies of SjR2 per egg to be determined. mRNA was extracted from *S. japonicum* eggs belonging to different eggs types deposited in intestinal tissues from mice 45 days post-infection (Figure 3). Based on this analysis, the mean mRNA copy number per egg could be deduced to be 20.9 ± 3.56 copies.

#### Detection of mRNA from *S. japonicum* eggs in liver and colon tissues from *S. japonicum* infected mice

**Fig. 4:**
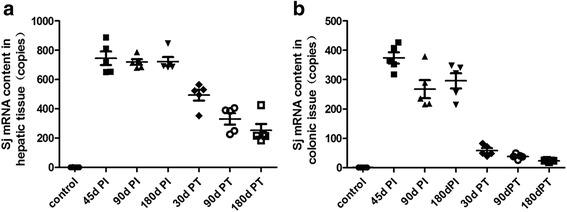
mRNA levels of *S. japonicum* eggs in liver and intestinal tissues from mice at different time points either post infection or post treatment. mRNA of *S. japonicum* eggs deposited in intestinal and liver tissues in 45dPI,90dPI,180dPI and 30dPT groups were more significantly than that of the normal group respectively (*P* < 0.001). mRNA of *S. japonicum* eggs in livers were more significantly than that of in intestines at every time point of post infection (*P* < 0.05)

SjR2 mRNA was undetectable in the liver and colon tissue samples from uninfected control mice (Table 6 and Figure 4). Compared with control mice, SjR2 mRNA levels in the intestine and liver tissue from 45 dPI, 90 dPI, 180 dPI and 30 dPT groups were highly significantly different (*P*<0.001). However, there was no difference between the SjR2 mRNA levels in the 45 dPI, 90 dPI, 180 dPI and 30 dPT groups (*P*>0.05). In addition, the mRNA levels of *S.japonicum* eggs in livers were higher than those in intestines at every infection time point (*P*<0.05).

**Table 6:** Comparison of the average mRNA levels (copies) of eggs in liver and intestinal tissues collected from mice at different time points either post infection or post treatment

Group	Control	45dPI	90dPI	180dPI	30dPT	90dPT	180dPT
Egg mRNA in liver	0.59 ±0.27	744.74 ±103.18*	718.73 ±42.71*	722.74 ±69.94*	493.43 ±82.35*	330.80 ±86.06*	252.59 ±97.43*
Egg mRNA in colon	0.68 ±0.14	374.22 ±43.50**	267.49 ±68.98**	296.04 ±58.12**	58.25 ±18.02**	38.06 ±8.22**	23.11 ±2.75**

*: *P* = 0000, comparison mRNA of *S. japonicum* eggs in liver tissues from each infected mouse group with that of control mice

**: *P* < 0.01, comparison of mRNA of *S. japonicum* eggs in colon tissues with that in liver at the same time point

The mRNA levels of *S. japonicum* eggs deposited in intestinal and liver tissues in the 45dPI,90dPI,180dPI and 30dPT groups were significantly higher than that of the normal group, respectively (*P* < 0.001). The mRNA levels of *S. japonicum* eggs in livers were more significantly higher than those in the intestines at every post infection time point (*P* < 0.05).

#### Detection of mRNA in different morphological egg types retained in biopsy samples of rectal mucous membrane from *S. japonicum* patients

**Fig. 5:**
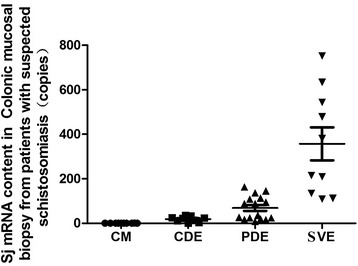
*S. japonicum* specific mRNA content in colonic mucosal biopsy from patients with suspected schistosomiasis. CM (colonic mucosal without eggs), CDE (completely degraded eggs in colonic mucosal), PDE (partially degraded eggs in colonic mucosal), SVE (suspected viable eggs in colonic mucosal)

Our findings from biopsy samples collected from 45 patients complement and confirm the results obtained from the experimental infections (Fig. 5; Table 7). Overall, the level of *S. japonicum* egg mRNA in colon tissue without eggs was significantly lower than in tissue containing completely degraded, partially degraded and suspected viable egg types (*P <* 0.001). Moreover, the level of mRNA in intestinal mucosa samples only containing completely degraded eggs was significantly lower than those containing partially degraded and suspected viable eggs (*P <* 0.01). The level of mRNA in the samples mainly containing suspected viable eggs was significantly higher than samples with partially degraded eggs (*P <* 0.01). As a diagnostic method to detect viable eggs the SjR2 PCR assay proved to be highly sensitive. Patient samples containing suspected viable eggs or partially degraded eggs had 100% or 50% positivity rates, respectively. Moreover, the results of detection of the samples either with completely degraded eggs or without any eggs were negative.

**Table 7:** The amount of *S. japonicum* egg mRNA and the positive rate of different types of eggs tested in intestinal mucosal biopsies from 45 patients determined by real time PCR

	CM cases	CME cases	SVE cases	Cases with mainly PDE	Only CDE cases
Tested Patients(n)	10	35	10	16	9
*S. japonicum* egg mRNA(M + SD)	0.50 ±0.17	135.37 ±189.62	357.04 ±234.84	69.26 ±62.13	19.17 ±11.06
*Positive patients(n)	0	18	10	8	0
Positivity rate (%)	0.00	51.43	100.00	50.00	0.00

CM (colonic mucosal without eggs), CME (colonic mucosal with eggs), SVE **(**suspected viable eggs in colonic mucosal), PDE (partially degraded eggs in colonic mucosal), CDE (completely degraded eggs in colonic mucosal)

* Content of positivity: the mRNA content of a sample with eggs was double that of viable eggs (20.9 ± 3.56 copies)

## Discussion

Schistosome egg viability, from a biological perspective, refers to the survival of eggs after being laid by a female worm till egg death. The main factor affecting viability is the micro environment encountered by the egg. For example, eggs deposited in living host tissues can lead to a short survival time (generally for one month, up to three months) due to the effect of the host immune system. Eggs trapped in feces outside the mammalian host can survive for 80 days at 1–4 °C [35] whereas eggs preserved in purified water at 4 °C can still hatch after 196 days [36].

Survival times of schistosome eggs are measured using the miracidium hatching method for eggs in feces and morphological observation of eggs in tissue samples. However, according to our findings, assessing schistosome egg viability using morphological observation of sigmoid-rectal biopsy samples is inadequate. Morphological diagnosis is not ideal for two reasons: microscopic evaluation requires a specialized and experience operator; and it is very difficult to determine the viability of partially degraded or/and completely degraded eggs within colon biopsy tissue, especially atypical eggs with indeterminable viability that are easily misdiagnosed.

At present, although there are many clinical diagnostic and field monitoring methods for schistosomiasis, including immunological detection of specific antigens or antibodies and molecular biological measurement of specific gene fragments, the gold standard for definitive diagnosis is microscopic observation of eggs or miracidia in stool or intestinal tissues for *S. japonicum* and *S. mansoni* and urine or bladder tissues for *S. haematobium*. However, the sensitivities of the Kato-Katz thick smear technique and miracidium hatching test do not exceed 70% [37, 38], and rectal examination results commonly yield false positive results for *S. japonicum* and *S. mansoni* [39].

We believe that the development of a rapid and simple method for determining the viability of schistosome eggs will help to further improve the effectiveness of rectal examination in the etiological diagnosis of schistosomiasis. In the present study, we evaluated several biomarkers and undertook morphological observations in the mouse model of schistosome infection and with schistosomiasis patients. Our results showed that eggs that were positive using a biomarker assay could be assumed to be viable. Eggs definitively confirmed by morphology to be viable, including immature and mature eggs, were positive using a biomarker assay; for example, the SDHG assay had a positivity rate of 97–99%. In addition, our biomarker assays generated positive results from difficult to diagnose samples that contained partially denatured, suspected viable or unknown type eggs.

In summary, this study had the following findings. Within infected mouse liver and intestinal tissues collected one month post PZQ treatment, few eggs were determined to be viable morphologically compared with the increased number of viable eggs detected with biomarkers. Furthermore, three months post treatment, mouse liver and intestinal tissue had no morphologically viable eggs but SDHG-positive eggs were still detected. These results indicate that a proportion of schistosome eggs can survive for about three months in the mouse model after parasite clearance.

Unlike the simple infection pattern in the mouse model, samples from schistosomiasis japonica patients were more complex. Morphological observation of the eggs in biopsied colonic mucosa from 70 cases of suspected schistosomiasis japonica patients indicated that repeated contact with infectious water and PZQ treatment can make definitive diagnosis difficult. In particular, for some cases there was no direct relationship between existing viable eggs and PZQ treatment. Following clinical proctoscopy, all 70 cases were reported to have “recent and long-term degraded eggs” due to the difficulty in identifying egg viability. However, by using biomarkers assays, such as egg-specific mRNA, 50% of cases with suspected viable eggs were found to have viable eggs compared with 10% of cases using morphology alone. In addition, from 30 suspected schistosomiasis cases, 13 were found to have viable eggs when using SDHG as a biomarker. However, the major benefit in using biomarker assays will be determining schistosome egg viability in colon biopsy tissue samples containing unknown type and partially degraded eggs.

Among the tested biomarkers there was no significant difference in mouse tissues before treatment for three biomarkers (CalS and AOS and SDHG). However, the sensitivity of AOS and SDHG were lower than that of ALP. Nonetheless, ALP, AOS, SDHG and CalS were significantly higher than the morphological observations. The highest sensitivity was obtained with SDHG detection and the MTT method and SjR2 mRNA detection with RT-qPCR. These two methods may prove to be more effective in the clinic for evaluating *S. japonicum* egg viability than the conventional morphological procedure. The tested biomarkers were effective for samples containing partially degraded, suspected viable and unknown type eggs in the mouse model post-treatment and for suspected schistosomiasis patients. Both SDHG and AOS assays are simple and convenient for detecting egg viability within host tissue but SDHG was more sensitive.

This is the first study reporting the use of four new biomarkers (CalS, AOS, SDHG and SjR2 mRNA) for measuring the viability of *S. japonicum* eggs. All four biomarkers have value for differentiating between viable and non-viable schistosome eggs. In particular, CalS, SDHG and SjR2 mRNA all offer advantages over morphological observation. However, each method has its advantages and disadvantages in the context of clinical practice. For example, the CalS detection method, similar to ALP detection, has high sensitivity, but requires a paraffin or frozen section of the test sample, adding complexity and possibly influencing the results because of egg loss during the procedure. Applying the real-time fluorescent quantitative PCR method to detect the specific mRNA of *S. japonicum* eggs is a sensitive, specific and quantitative evaluative method, but the technical requirements will restrict its wider use in the clinic and field. Both DAB detection of AOS and the MTT detection of SDHG have the advantage of a simple, one step operation and rapid results (within 30 min) for detecting viable eggs in colonic biopsy specimens. It should be noted that for the AOS or SDHG assays tissue blocks need to be fully compressed between two slides to effectively expose the eggs before testing. In addition, for correctly assessing weak positive eggs a comparison must be made with the unstained egg as the background color may affect the results.

## Conclusions

This paper reports on the use of four new markers (CalS, AOS, SDHG and SjR2 mRNA) for assessing the viability of schistosome eggs. The results of our morphological observations indicated that in schistosomiasis hosts, especially human patients, the morphology of schistosome eggs is varied and complex, and clinical diagnosis is particularly difficult for samples containing partially degraded eggs, suspected viable eggs or unknown type eggs, whereas all four biomarkers we measured were able to differentiate between viable and non-viable schistosome eggs and detect a high proportion of viable eggs in samples with partially degraded eggs, suspected viable or unknown type eggs. Among the four biomarkers, the detection of SjR2 mRNA was the most specific and sensitive method, while the SDHG method is simple, rapid and clinically practical. Our findings suggest that, compared with conventional diagnosis by endoscopic biopsy using egg morphology, SDHG is able to accurately determine the viability of schistosome eggs regardless of morphology type. Therefore, SDHG is the most practical for clinical application and would improve the accuracy of the diagnosis of active schistosome infection.

## Additional files

Additional file 1: Figure S1. Categories of eggs in colon tissue from infected mice. A: Immature eggs (smaller with embryonic cells present); B, D, I: Unknown viability eggs; C and E: Mature eggs (larger in size with miracidium present); F: Partially degraded eggs (miracidium with disordered structure with the the appearance of black particles); G and H: Completely degraded eggs (black particles present in eggs or whole egg appears black). Magnification is 100× for all images. (JPEG 52 kb)

Additional file 2: Figure S2. Types of eggs in biopsied colonic mucosa from schistosomiasis patients. Viable eggs with an intact miracidium are present in panels A, B and H (red arrows). Panels C, D and L contain partially degraded eggs that are light in color with disordered structure (green arrows). Panels B, G, I, J, K, M and N reveal completely degraded black eggs (black arrows). Unknown viability eggs containing miracidia with unclear structure are present in panels A, E, F and O (yellow arrows). Images are all at 100× magnification. (JPEG 88 kb)

Additional file 3: Table S1. Clinical data and egg viability in proctoscopic tissue samples collected from 76 schistosomiasis patients. (DOCX 12 kb)

Additional file 4: Figure S3. ALP staining results for *S. japonicum* eggs in mouse colonic tissue. A: 45dPI group; B: 90dPI group; C: 180dPI group; D:30dPT group; E: 90dPT group; F: 180dPT group. The arrows in Fig. A point to positive eggs, which were stained blue/black by NBT. The staining results show that: all eggs in the 45dPI group were positive and negative eggs began to appear in the 90dPI group; Positive eggs decreased after treatment of mice with PQZ and, notably, eggs in the180dPI group were all negative. The eggs that had no color development were mostly empty or had a fuzzy structure (400× magnification). (JPEG 60 kb)

Additional file 5: Figure S4. CalS staining results for *S. japonicum* eggs in mouse colonic tissue. A: 45dPI group; B: 90dPI group; C: 120dPI group; D: 30dPT group; E: 90dPT group; F: 180dPT group. Eggs indicated by the blue arrows were negative (live) and stained blue with the von Kossa stain. Calcified eggs, were positively stained black? with the von Kossa stain and are highlighted (red arrows). In the Figure, as the tissue background was stained with eosin, the miracidium in the egg was also stained red (400× magnification). (JPEG 62 kb)

Additional file 6: Figure S5. AOS staining results for *S. japonicum* eggs in mouse colonic tissue. A, B and C indicate free eggs isolated from either tissues of infected mice with *S. japonicum* at 45 days and 180 days post infection or tissues of treated mice at 90 days post treatment. After staining with DAB, eggs in Fig. A developed a positive deep yellow or brown color. Some eggs in Fig. B were positive response and are indicated by blue arrows. In Fig. C, no eggs were positive. Figs 1, 2, 3, 4, 5 and 6 (1: 45 dPI group; 2: 90 dPI group; 3: 120 dPI group; 4: 30 dPT group; 5: 90 dPT group; 6: 180 dPT group) show DAB stained eggs in colon tissue from mice. Among them, all eggs in Figs 1 and 2 developed a positive deep yellow color. Some eggs in Figs 3 and 4 were positive indicated by blue arrows. None of the eggs in Figs 5 and 6 were positive. Eggs that were negative were morphologically empty and had a fuzzy structure or appeared black (400× magnification). (JPEG 82 kb)

Additional file 7: Figure S6. Amplified, standard and melt curve lines of *S. japonicum*-specific RNA real-time qPCR. The first 6 standards of the real-time qPCR (copy number was 10^5^ Copies ~ 1 Copy) presented as a complete amplified curve, but the last standard (0.1 copy) did not have a t completely amplified curve. The linear relationship of the standard curve line was optimum, and the amplifying effectiveness was 98.05%; the dissolved melt curve line presented as single peak. (JPEG 50 kb)

## Abbreviations

ALP: Alkaline phosphatase
AOS: Antioxidase
BCIP: 5-Bromo-4-chioro-3- indolyl-phosphate
CalS: Calcified substance
CDE: Completely degraded eggs in colonic mucosal
cDNA: Complementary DNA
CM: Colonic mucosal without eggs
DAB: 3,3-dimethylbenzidine
DEPC: Diethyl pyrocarbonate
ECCPR: Ethical Committee of Center for Parasitology Research
mRNA: Messenger RNA
MTT: Methyl Thiazolyl Tetrazolium
NBT: Nitro-Blue-Tetrazolium
ORF: Open reading frame
PDE: Partially degraded eggs in colonic mucosal
PI: Post infection
PT: Post treatment
PZQ: Praziquantel
RT: Room temperature
RT-qPCR: Fluorescence quantitative PCR
*S. japonicum*: *Schistosoma japonicum*
SDHG: Succinic dehydrogenase
SjR2: Retrotransposons 2 of *S.japonicum* genome
SVE: Suspected viable eggs in colonic mucosal
