# Supplementary figures and images for: Clinical diagnostic value of viable Schistosoma japonicum eggs detected in host tissues
Source: BMC Infect Dis. 2017 Apr 4;17:244. doi: 10.1186/s12879-017-2362-4 (PMC5379624; doi:10.1186/s12879-017-2362-4)

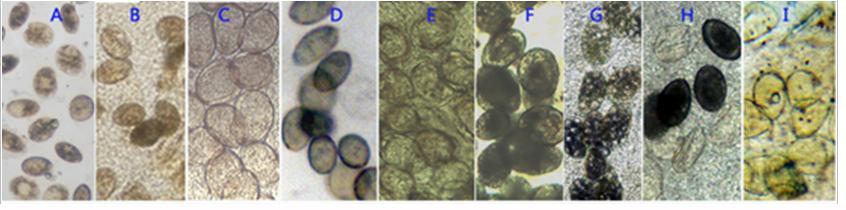

Supplement: Supplementary file 1 — Categories of eggs in colon tissue from infected mice. A: Immature eggs (smaller with embryonic cells present); B, D, I: Unknown viability eggs; C and E: Mature eggs (larger in size with miracidium present); F: Partially degraded eggs (miracidium with disordered structure with the the appearance of black particles); G and H: Completely degraded eggs (black particles present in eggs or whole egg appears black). Magnification is 100× for all images. (JPEG 52 kb) [file 12879_2017_2362_MOESM1_ESM.jpg]

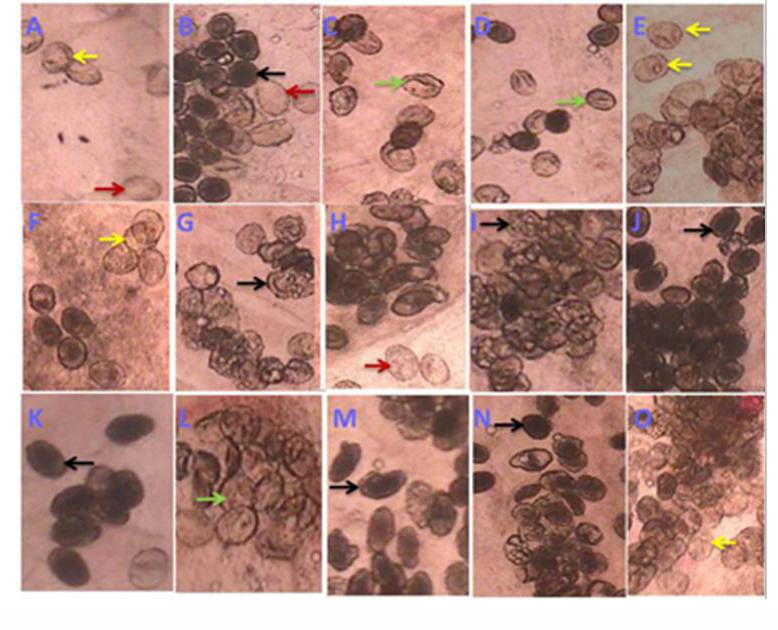

Supplement: Supplementary file 2 — Types of eggs in biopsied colonic mucosa from schistosomiasis patients. Viable eggs with an intact miracidium are present in panels A, B and H (red arrows). Panels C, D and L contain partially degraded eggs that are light in color with disordered structure (green arrows). Panels B, G, I, J, K, M and N reveal completely degraded black eggs (black arrows). Unknown viability eggs containing miracidia with unclear structure are present in panels A, E, F and O (yellow arrows). Images are all at 100× magnification. (JPEG 88 kb) [file 12879_2017_2362_MOESM2_ESM.jpg]

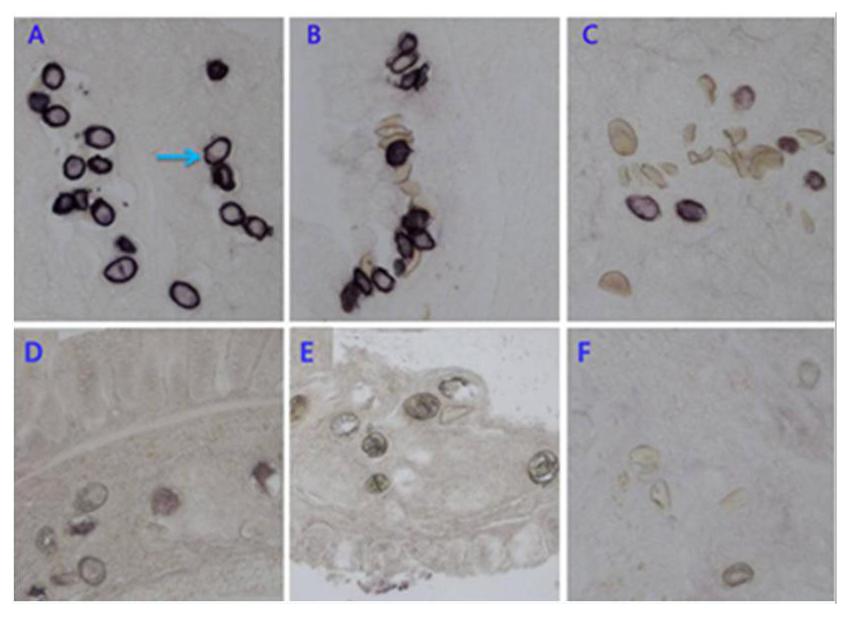

Supplement: Supplementary file 4 — ALP staining results for S. japonicum eggs in mouse colonic tissue. A: 45dPI group; B: 90dPI group; C: 180dPI group; D:30dPT group; E: 90dPT group; F: 180dPT group. The arrows in Fig. A point to positive eggs, which were stained blue/black by NBT. The staining results show that: all eggs in the 45dPI group were positive and negative eggs began to appear in the 90dPI group; Positive eggs decreased after treatment of mice with PQZ and, notably, eggs in the180dPI group were all negative. The eggs that had no color development were mostly empty or had a fuzzy structure (400× magnification). (JPEG 60 kb) [file 12879_2017_2362_MOESM4_ESM.jpg]

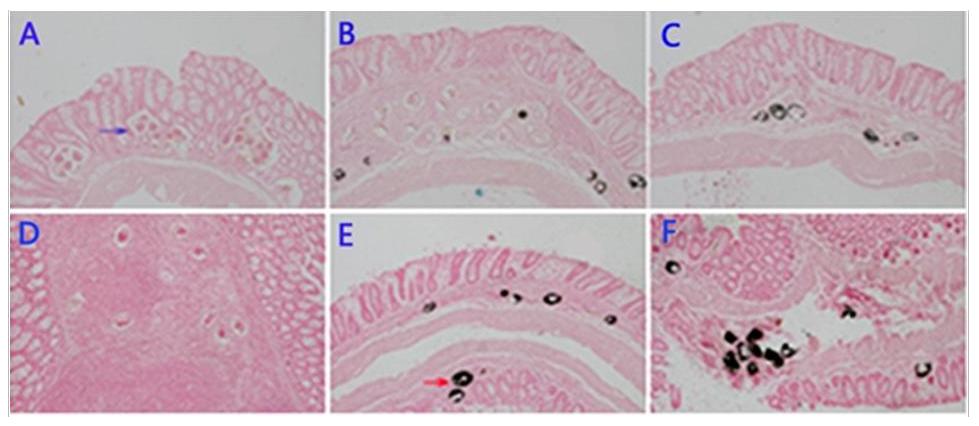

Supplement: Supplementary file 5 — CalS staining results for S. japonicum eggs in mouse colonic tissue. A: 45dPI group; B: 90dPI group; C: 120dPI group; D: 30dPT group; E: 90dPT group; F: 180dPT group. Eggs indicated by the blue arrows were negative (live) and stained blue with the von Kossa stain. Calcified eggs, were positively stained black? with the von Kossa stain and are highlighted (red arrows). In the Figure, as the tissue background was stained with eosin, the miracidium in the egg was also stained red (400× magnification). (JPEG 62 kb) [file 12879_2017_2362_MOESM5_ESM.jpg]

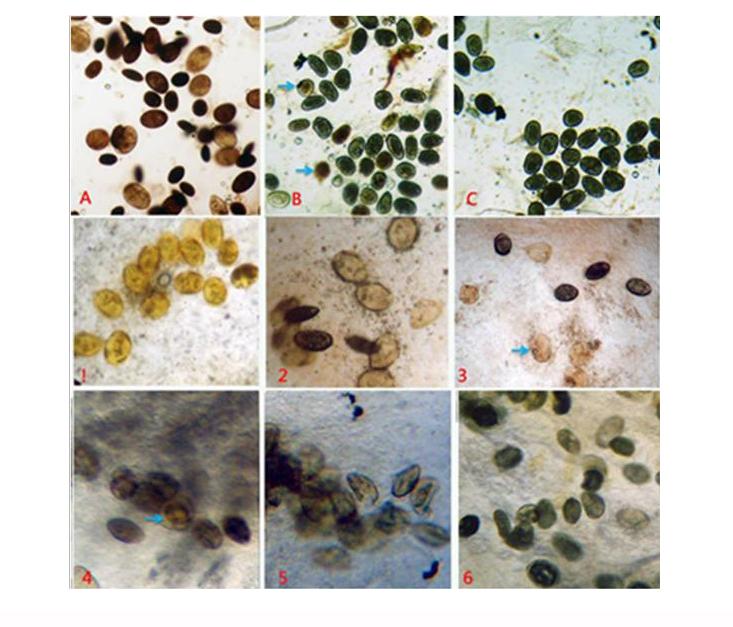

Supplement: Supplementary file 6 — AOS staining results for S. japonicum eggs in mouse colonic tissue. A, B and C indicate free eggs isolated from either tissues of infected mice with S. japonicum at 45 days and 180 days post infection or tissues of treated mice at 90 days post treatment. After staining with DAB, eggs in Fig. A developed a positive deep yellow or brown color. Some eggs in Fig. B were positive response and are indicated by blue arrows. In Fig. C, no eggs were positive. Figs 1, 2, 3, 4, 5 and 6 (1: 45 dPI group; 2: 90 dPI group; 3: 120 dPI group; 4: 30 dPT group; 5: 90 dPT group; 6: 180 dPT group) show DAB stained eggs in colon tissue from mice. Among them, all eggs in Figs 1 and 2 developed a positive deep yellow color. Some eggs in Figs 3 and 4 were positive indicated by blue arrows. None of the eggs in Figs 5 and 6 were positive. Eggs that were negative were morphologically empty and had a fuzzy structure or appeared black (400× magnification). (JPEG 82 kb) [file 12879_2017_2362_MOESM6_ESM.jpg]

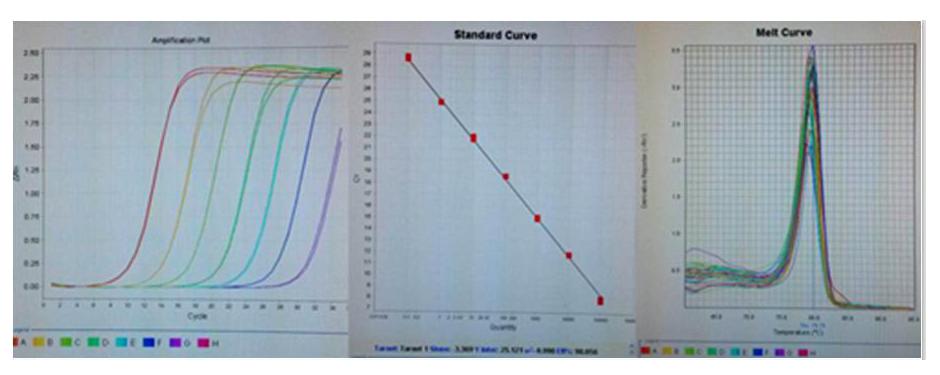

Supplement: Supplementary file 7 — Amplified, standard and melt curve lines of S. japonicum-specific RNA real-time qPCR. The first 6 standards of the real-time qPCR (copy number was 105 Copies ~ 1 Copy) presented as a complete amplified curve, but the last standard (0.1 copy) did not have a t completely amplified curve. The linear relationship of the standard curve line was optimum, and the amplifying effectiveness was 98.05%; the dissolved melt curve line presented as single peak. (JPEG 50 kb) [file 12879_2017_2362_MOESM7_ESM.jpg]
