# Supplementary material for: Clinical diagnostic value of viable Schistosoma japonicum eggs detected in host tissues
Source: BMC Infect Dis. 2017 Apr 4;17:244. doi: 10.1186/s12879-017-2362-4 (PMC5379624; doi:10.1186/s12879-017-2362-4)
Supplement: Supplementary file 3 — Clinical data and egg viability in proctoscopic tissue samples collected from 76 schistosomiasis patients. (DOCX 12 kb) [file 12879_2017_2362_MOESM3_ESM.docx]

**Supplementary table one: Clinical data and egg viability in proctoscopic tissue samples collected from 76 schistosomiasis patients**

|  | Cases | Viable eggs | Unknown viability eggs | Partially degraded eggs | Completely degraded eggs | Only completely degraded eggs |
| --- | --- | --- | --- | --- | --- | --- |
| Case numbers | 76 | 44 | 32 | 69 | 73 | 9 |
| Male | 46 | 24 | 20 | 44 | 46 | 7 |
| Female | 20 | 20 | 12 | 25 | 27 | 2 |
| History of contact with infectious water | 76 | 43 | 30 | 69 | 73 | 9 |
| Without treatment | 14 | 11 | 7 | 14 | 14 | 0 |
| 1-3 years after treatment | 29 | 21 | 9 | 25 | 27 | 4 |
| Over 3 years after treatment | 33 | 12 | 16 | 30 | 32 | 5 |
| Diarrhea symptoms | 68 | 40 | 29 | 63 | 64 | 6 |
| Ultrasound B suggests hepatic disease due to schistosomiasis | 23 | 15 | 7 | 22 | 22 | 0 |
